# Bacterial membrane vesicles: orchestrators of interkingdom interactions in microbial communities for environmental adaptation and pathogenic dynamics

**DOI:** 10.3389/fimmu.2024.1371317

**Published:** 2024-03-21

**Authors:** Lijun Xiu, Yuwei Wu, Gongshi Lin, Youyu Zhang, Lixing Huang

**Affiliations:** ^1^ State Key Laboratory of Mariculture Breeding, Fisheries College of Jimei University, Xiamen, Fujian, China; ^2^ Xiamen Marine & Fisheries Research Institute, Xiamen, Fujian, China; ^3^ Institute of Electromagnetics and Acoustics, School of Electronic Science and Engineering, Xiamen University, Xiamen, Fujian, China

**Keywords:** bacteria, membrane vesicles, environmental adaptation, pathogenic process, intermicrobial interactions

## Abstract

Bacterial membrane vesicles (MVs) have attracted increasing attention due to their significant roles in bacterial physiology and pathogenic processes. In this review, we provide an overview of the importance and current research status of MVs in regulating bacterial physiology and pathogenic processes, as well as their crucial roles in environmental adaptation and pathogenic infections. We describe the formation mechanism, composition, structure, and functions of MVs, and discuss the various roles of MVs in bacterial environmental adaptation and pathogenic infections. Additionally, we analyze the limitations and challenges of MV-related research and prospect the potential applications of MVs in environmental adaptation, pathogenic mechanisms, and novel therapeutic strategies. This review emphasizes the significance of understanding and studying MVs for the development of new insights into bacterial environmental adaptation and pathogenic processes. Overall, this review contributes to our understanding of the intricate interplay between bacteria and their environment and provides valuable insights for the development of novel therapeutic strategies targeting bacterial pathogenicity.

## 1 Introduction

The concept of bacterial membrane vesicles (MVs) can be traced back to the 1960s when vesicles derived from bacterial cells were first observed in *Escherichia coli* and *Vibrio cholerae*, and it was proposed through electron microscopy that these vesicles originated from the outer membrane of bacteria (1, 2). However, due to limited understanding of their specific biological functions at that time, these small vesicles were considered as by-products of bacterial growth imbalance and metabolic remnants. Recent studies have demonstrated that bacterial MVs play important roles in bacterial physiology and pathogenesis and have thus attracted widespread research attention from scientists.

**Figure 1:**
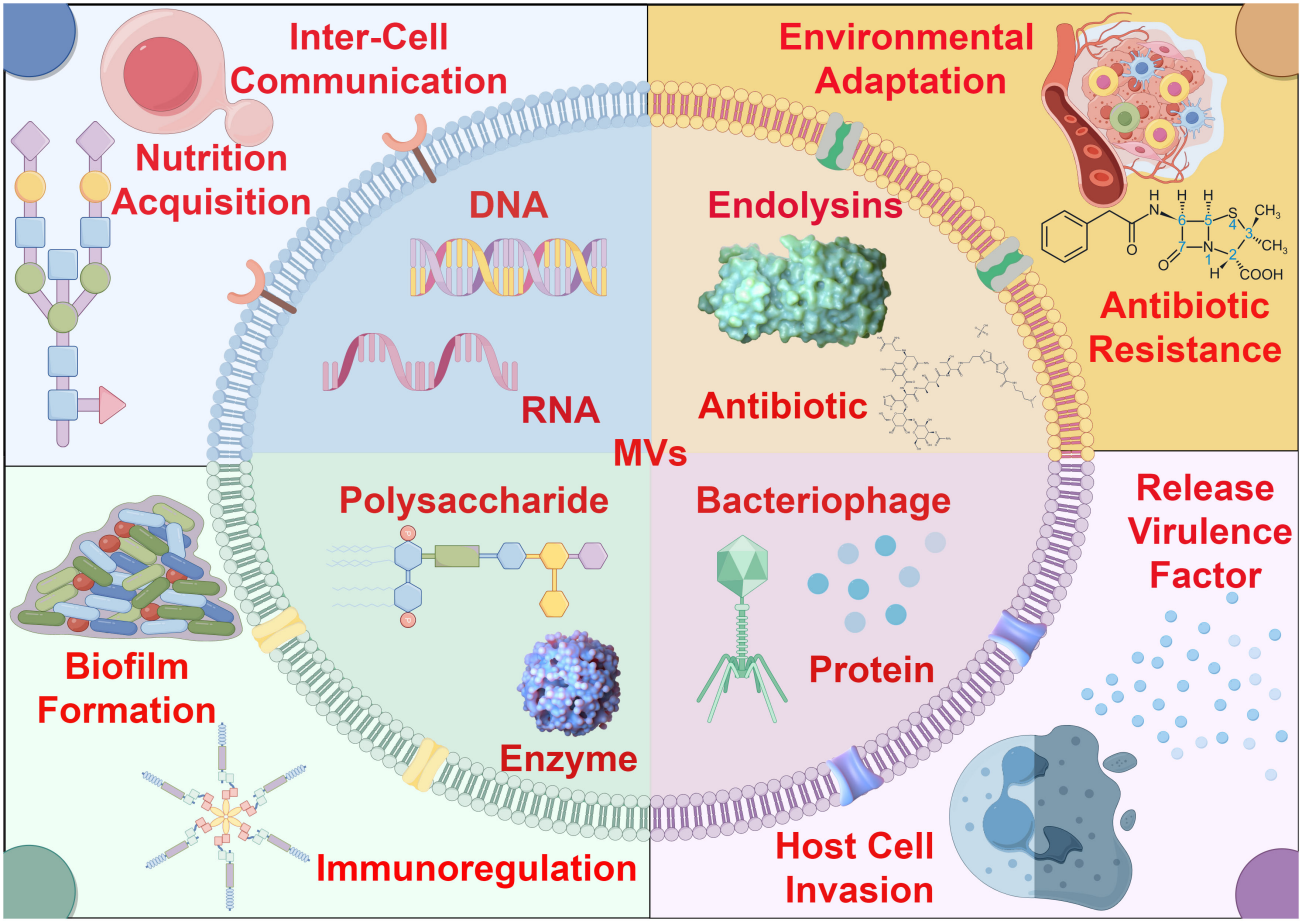
The role of bacterial membrane vesicles in environmental adaptation and pathogenic dynamics. Bacterial MVs exhibit a bilayer membrane structure, and their surfaces may carry specific receptors or membrane proteins. Due to differences in bacterial physiological conditions and environmental stress, various types of MVs are actively secreted, carrying and transporting a diverse range of biomolecules, including nucleic acids, proteins, enzymes, antibiotics, bacteriophages and so on. These MVs with distinct contents play crucial roles in microbial communities for environmental adaptation and pathogenic dynamics.

Bacterial membrane vesicles (MVs) represent a category of spherical vesicles with diameters ranging from 10 to 400 nanometers, characterized by a bilayer lipid membrane structure derived from the bacterial cell membrane release process. These vesicles exhibit unique and multifunctional secretion patterns and transport mechanisms. Typically, specific cargo is packaged and transported within or on the surface of MVs. MVs of varying contents participate in diverse physiological activities of bacteria, including nutrient acquisition, intercellular communication, environmental adaptation, antibiotic resistance, release of virulence factors, invasion of host cells, biofilm formation, and immune modulation, thereby playing adaptable roles based on contextual requirements (**Figure 1**). These bacterial MVs are widely present in various bacterial species, being secreted not only by Gram-negative and Gram-positive bacteria (3), but also originating from cell lysis induced by endolysins, which explains why bacteria can produce different types of MVs. Microorganisms from different taxa can release extracellular vesicles that are associated with the cell membrane into the surrounding environment, which explains their high abundance in natural environments such as soil and oceans, as well as their presence in various internal environments like host tissues, thereby playing various functional roles in the microbial ecosystem (4).

**Figure 2:**
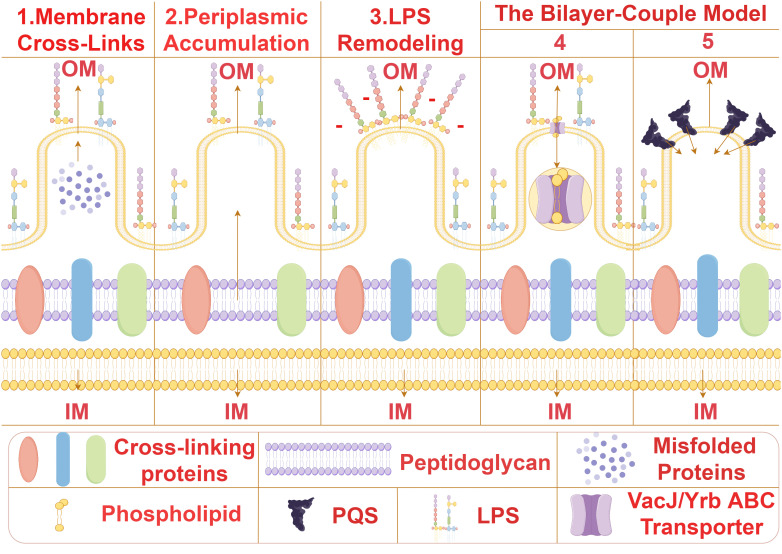
The biogenesis mechanisms of MVs. Several possible biogenesis mechanisms of bacterial MVS from different species have been proposed. (1) Membrane Cross-Links:Removal of proteins anchoring the outer membrane and underlying peptidoglycan enhances membrane fluidity, allowing the membrane to bend and form membrane vesicles (MVs) (5–8). (2) Periplasmic Accumulation:Local aggregation of peptidoglycan fragments or misfolded proteins in the periplasm induces curvature of the outer membrane (9–11). (3) Lipopolysaccharide Remodeling:Due to charge repulsion, locally enriched lipopolysaccharide (LPS) species with anionic charges induce curvature of the outer membrane, leading to subsequent formation of MVs (12–14). The Bilayer-Couple Model. (4). Downregulation of the VacJ/Yrb ABC transporter involved in retrograde transport of phospholipids in the outer membrane results in the accumulation of phospholipids in the outer leaflet of the outer membrane. This accumulation causes rapid expansion of the outer leaflet compared to the inner leaflet, resulting in membrane bending and MV formation (15, 16). (5) Bacteria secrete Pseudomonas quinolone signal (PQS) into the extracellular space, which subsequently inserts into the outer leaflet of the outer membrane through interactions with lipids and phospholipids. The insertion of PQS into the outer membrane causes expansion of the outer leaflet, increasing the generation of MVs (17–19).

Bacterial MVs formation is initiated by the expansion and contraction of the bacterial outer membrane, resulting in the release of spherical structures containing cytoplasmic proteins, lipids, and nucleic acids. The biogenesis of MVs is a complex and dynamic process involving various intricate molecular mechanisms such as membrane bending, peptidoglycan remodeling, and lipid and protein aggregation. At present, the universal mechanism of biogenesis of bacterial MVs has not been confirmed, and several possible biogenesis mechanisms of bacterial MVS from different species have been proposed (**Figure 2**). (1) The removal of proteins anchoring the outer membrane and the underlying peptidoglycan serves to augment membrane fluidity, thereby enabling the membrane to flex and give rise to membrane vesicles (MVs) (5–8). For instance, in *Pseudomonas aeruginosa*, proteins anchoring the outer membrane impacts MVs production. Inactivation of lpp protein can results in increased MVs production (6).This orchestrated modification in membrane dynamics represents a pivotal step in the biogenesis of MVs, emphasizing the significance of protein-lipid interactions in membrane curvature and vesicle formation. (2) The localized aggregation of peptidoglycan fragments or misfolded proteins in the periplasmic space initiates a curvature phenomenon in the outer membrane of bacterial cells (9–11).This mechanism is proposed for Porphyromonas gingivalis (10), This intriguing process underscores the intricate interplay between molecular components in the periplasm and the resulting morphological changes in the outer membrane. (3) Owing to charge repulsion, locally enriched lipopolysaccharide (LPS) species possessing anionic charges induce curvature of the outer membrane, culminating in the subsequent formation of MVs (12–14). From this mechanism, we can reasonably speculate that MVs may be formed in the position where LPS is more abundant, and the bending of OM may alleviate the charge repulsion in the region (12). The electrostatic forces governing this curvature offer a nuanced perspective on the physicochemical principles guiding membrane architecture and vesiculation. (4) The downregulation of the VacJ/Yrb ABC transporter, a key player in the retrograde transport of phospholipids in the outer membrane, leads to the accumulation of phospholipids in the outer leaflet (15, 16). This accumulation results in the rapid expansion of the outer leaflet relative to the inner leaflet, instigating membrane bending and facilitating the formation of MVs. The intricate regulation of phospholipid transport elucidates a molecular mechanism underlying membrane curvature dynamics. (5) Bacteria release the Pseudomonas quinolone signal (PQS) into the extracellular milieu, where it subsequently inserts into the outer leaflet of the outer membrane through interactions with lipids and phospholipids (17–19). This insertion of PQS induces expansion of the outer leaflet, thereby amplifying the generation of MVs. The interplay between bacterial signaling molecules and membrane dynamics adds a layer of complexity to our understanding of MV biogenesis and highlights the multifaceted nature of bacterial communication and vesicle formation.

In Gram-negative bacteria, MVs are formed primarily through two distinct avenues (20): one is through outer membrane blebbing (B-type MVs), and the other is through explosive cell lysis or bubbling cell death (E-type MVs), resulting in the curling and self-assembly of shattered membrane fragments. Afterwards, B-type MVs can be subdivided into outer membrane vesicles (OMVs), Outer-inner MVs (OIMVs) and Cytoplasmic Membrane Vesicles (CMVs) according to the different biological modes and contents. Relatively, E-type MVs can also be subdivided into explosive cytochrome membrane veins (ECMVs), explosive outer membrane veins (EOMVs) and explosive outer–inner membrane veins (EOI MVS) (3).

Bacteria can release soluble substances and complexes through MVs, however, unlike other secretion mechanisms, MVs allow bacteria to also release insoluble molecules in a concentrated, protected, and targeted manner to distant targets. Key features of MVs biogenesis include the outward expansion of regions lacking membrane-peptidoglycan linkages, enhancing the ability of MVs biogenesis without compromising the integrity of the outer membrane, enrichment or exclusion of specific proteins and lipids, and the lack of requirement for ATP/GTP hydrolysis to provide energy for membrane division (21). Furthermore, MVs have been shown to originate not only from live cells but also from cell lysis induced by endolysins. In *Pseudomonas aeruginosa* biofilms, the explosive cell lysis of cell communities leads to the release of cytoplasmic contents and the rapid formation of MVs through the production of ruptured membrane fragments (22).

The biogenesis of bacterial MVs is regulated by several key factors, one of which is curvature-inducing proteins such as EspH, FtsZ, and TolQ. These proteins promote outer membrane deformation and neck-like structure formation, leading to the generation of vesicles. Another important factor is peptidoglycan remodeling enzymes, such as lytic transglycosylases, endolysins, and hydrolases. These enzymes can cleave the covalent bonds between peptidoglycan and the outer membrane, thereby facilitating vesicle secretion and release. For example, studies have shown that the formation of *Bacillus subtilis* MVs is triggered by the expression of endolysins in bacterial subpopulations, resulting in the formation of pores in the cell wall. Through these pores, the intracellular material protrudes from the cytoplasm into the extracellular space and is released as MVs (23). Due to the loss of membrane integrity, the triggered cells eventually die, while the bacteria releasing endolysins induce neighboring bacteria to form MVs.

Some studies have identified protein and lipid motifs involved in the biogenesis and classification of bacterial MVs, such as lipopolysaccharides (12), outer membrane pore proteins, and chaperone proteins. Additionally, the selective packaging of proteins and lipids into bacterial MVs is a highly selective process involving specific targeting and recognition signals.

For instance, the human oral pathogen *Porphyromonas gingivalis* possesses a selective mechanism for packaging proteins into MVs, leading to the preferential encapsulation of virulence factors into MVs (14), and the exclusion of numerous outer membrane proteins from the protein cargo. In this specific sorting mechanism, lipopolysaccharides play a crucial guiding role. Due to their unique properties such as stability, biocompatibility, and payload encapsulation, bacterial MVs have been developed for potential biotechnological and medical applications (24, 25), such as drug delivery vehicles (26–28), vaccine adjuvants (29, 30), and diagnostic markers.

This paper offers a comprehensive review of the role of bacterial MVs in environmental adaptation and pathogenic processes, with a particular emphasis on their potential functions in intermicrobial interactions. This review lays the groundwork for further exploration of the role of bacterial MVs in the pathogenic microbial world, offering novel research avenues for scientists studying bacterial MVs. The elucidation of the multifaceted roles of MVs presented in this review not only advances our understanding of bacterial adaptation and pathogenicity but also serves as a springboard for the development of innovative strategies for combating pathogenic infections.

## 2 Function of bacterial MVs in environmental adaptation

Bacterial MVs serve as pivotal mediators in a myriad of physiological and adaptive processes, including nutrient acquisition, intercellular communication, environmental stress responses, and the regulation of antibiotic resistance. Understanding the multifaceted roles of MVs in bacterial environmental adaptation is crucial for elucidating their impact on bacterial fitness and their contribution to microbial community dynamics. This knowledge is indispensable for comprehending the intricate interplay between bacteria and their environment, providing insights that are fundamental for ecological and clinical implications.

### 2.1 Nutrition acquisition

Bacterial MVs play an essential role in nutrient acquisition. Numerous studies have demonstrated that MVs-mediated substance transport represents a novel bacterial nutrient transfer pathway, to be considered as an additional and independent secretion system, Secretion system type zero (31). By releasing enzymes, proteins, lipids, and other components, bacterial MVs assist in acquiring nutrients from the external environment, thus enhancing the efficiency of nutrient utilization by bacteria. Bacterial MVs can clear and transport various nutrients, which participate in different forms of transport and regulate physiological processes, thereby modulating various bacterial physiological activities. Bacterial MVs achieve this by carrying vesicles containing nutrients, releasing these substances into the surrounding microbial environment, or transferring them between cells. For example, the MVs of *Streptomyces coelicolor* contain complex metabolic products. The study encompasses aspects related to antibiotics, vitamins, amino acids, proteins, and carbon metabolism components, with a particular focus on the identification of a total of 166 proteins within MVs involved in cellular metabolism/differentiation, molecular processing/transport, and stress response. These proteins play a crucial role in bacterial morphological and physiological differentiation functions (32). These proteins remain protected from degradation by MVs, even after treatment with proteinase K, indicating their specific localization within MVs. Another study discovered that the Antarctic bacterium *Pseudoalteromonas distincta* ANT/505 possesses a polysaccharide degradation-associated γ-protein and can produce outer membrane vesicles (OMVs) and vesicle chains (VCs) on both polysaccharide and non-polysaccharide carbon sources. Under carbohydrate culture conditions, the cell surface expresses a higher level of specific proteins, and in all growth conditions, proteins encoded by genomic regions associated with polysaccharide degradation can be detected in MVs and VCs samples (33). Polysaccharides, a major nutritional source for bacteria, significantly impact various physiological activities in bacteria. Bacteria utilize polysaccharide enzymes to break down polysaccharides into monosaccharides, entering metabolic pathways to provide energy support for bacterial growth and metabolic activities. MVs derived from symbiotic bacteria are rich in glycoside hydrolases, effectively degrading environmental polysaccharides. Bacterial polysaccharide metabolism not only influences their survival and adaptability but also allows them to regulate and utilize different types of polysaccharides as energy and carbon sources in response to environmental changes, thus maintaining the necessary nutrients for growth and metabolism. These MVs equipped with hydrolytic enzymes demonstrate the ability to degrade polymeric substrates of various non-human glycoside hydrolases, providing a source of cut polysaccharides for utilization by members of the bacterial community, including rod-shaped bacteria and other microbial constituents (34). Such functionality of MVs contributes to the bacterial community within the entire microbial population, revealing novel functions of bacterial MVs.

The nutrients present in bacterial MVs can provide energy and raw materials to neighboring bacteria, and to some extent, alter the composition and metabolism of the surrounding bacterial community. Furthermore, studies have demonstrated that the bacterium *Alteromonas macleodii* KS62 can release a large quantity of MVs containing hydrolytic enzymes into the surrounding seawater environment (35). These MVs facilitate cell wall hydrolysis and the utilization of red algal biomass, thereby increasing the concentration of nutrients available for absorption and utilization by the bacterial community in the matrix environment, consequently converting the carbon-rich red algal biomass into bioethanol. These findings suggest that bacterial MVs can optimize growth conditions and adapt to the environment by altering biomass and nutrient availability. Additionally, the nutrient acquisition function of bacterial MVs relies not only on their internal components but also on surface substances or receptors. These surface elements interact with the surrounding environment to procure nutrients (36). The role of MVs in acquiring iron is particularly important, especially for hydrophobic iron carriers, which are released and dispersed in the environment via MVs. For instance, *Mycobacterium tuberculosis* increases the production of MVs under iron limitation, and these MVs contain mycobactin, which serves as an iron donor, supporting the replication of iron-limited Mycobacteria (37). The *P. aeruginosa* gene *tseF* synergistically interacts with the iron acquisition system, leading to the encapsulation of the iron-bound *Pseudomonas* quinolone signal (PQS) within bacterial MVs via H3-T6SS secretion (38). Additionally, the interaction between TseF and the iron receptors FptA and porin protein OprF promotes the transport of iron-related MVs to bacterial cells. *Cupriavidus necator* secretes the lipopolysaccharide-binding effector protein TeoL via T6SS1, which binds preferentially to MVs in the extracellular environment through a mechanism mediated by lipopolysaccharide carried by MVs (39). This lipopolysaccharide-mediated mechanism allows bacteria to utilize MVs from different species, providing them with an advantage in iron acquisition, inter-bacterial competition, and gene transfer. Similarly, research has shown that bacterial MVs of *Dietzia* sp. are involved in capturing and transporting extracellular heme, particularly the recovery of heme from environmental heme proteins. Under iron-limiting conditions, MVs are enriched with heme-binding proteins, and the heme carried by MVs can be utilized by various species (40). These findings underscore the significant role of bacterial MVs in the acquisition and exchange of nutrients among bacteria.

**Figure 3:**
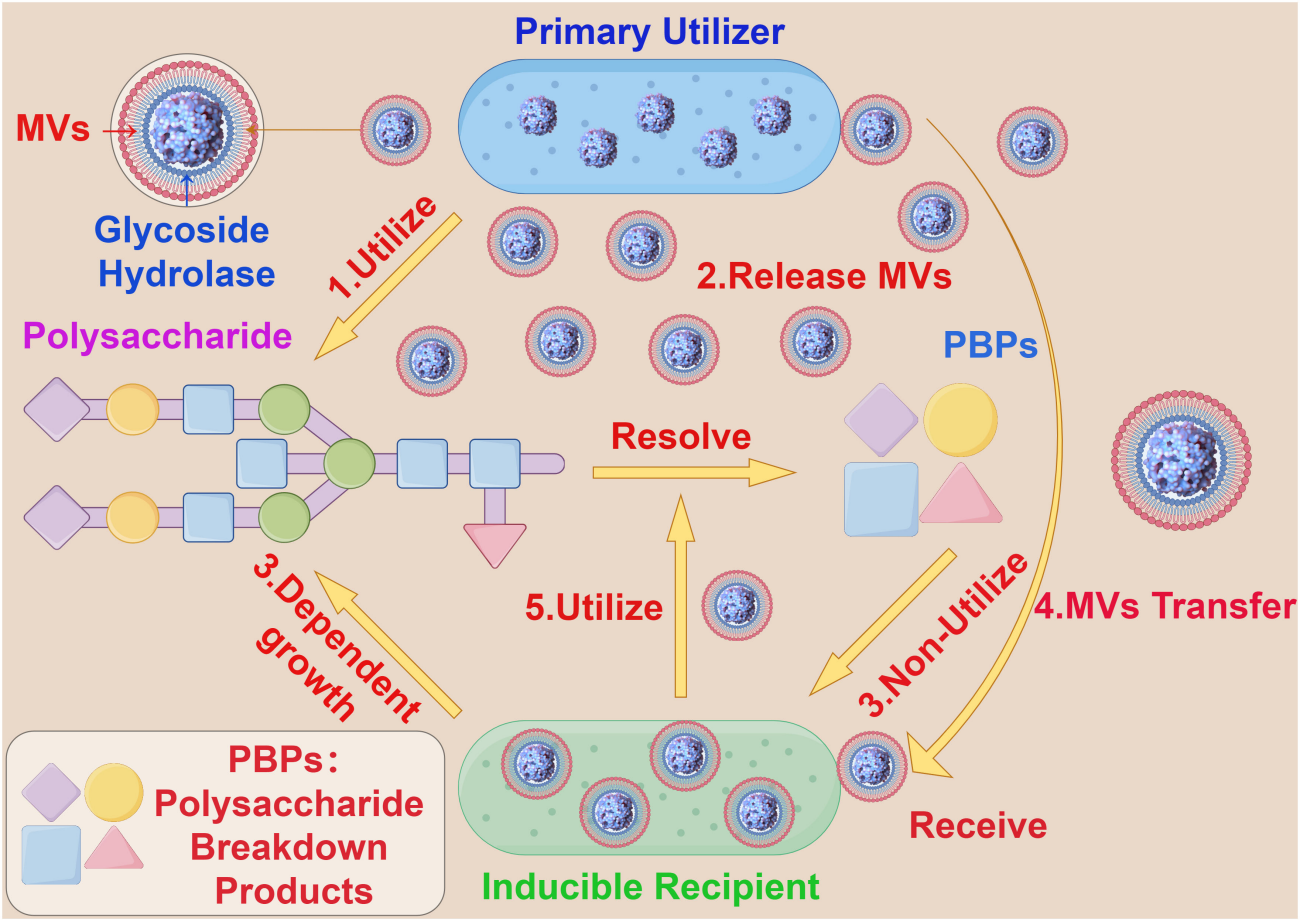
The interaction between bacterial MVs and bacteroides. (1) Primary Utilizer can decompose polysaccharide into PBPs by carbohydrate hydrolysis. (2) Primary Utilizer can release MVs containing Glycoside Hydrolase into the extracellular space. (3) Inducible Recipient depends on the growth of polysaccharide and cannot use PBPs. (4) The MVs containing Glycoside Hydrolase accepted by Inducible Recipient. (5) Inducible Recipient acquires the ability to decompose polysaccharides and is able to produce PBPs.

Additionally, bacterial MVs are involved in the competitive processes between bacterial populations by fusing with the outer membrane of other microorganisms, thereby killing other microbes in the environment to acquire and compete for nutritional components. For example, *Lysobacter spp* and *Myxococcus xanthus* can dissolve certain Gram-negative bacteria by releasing MVs containing active proteases, hydrolases, bacteriolytic enzymes and other secondary metabolites (41, 42). Interestingly, in the human gut, some coexisting *Bacteroides* release polysaccharide breakdown products (PBPs), which cannot be consumed by other species (receivers) that rely solely on polysaccharides for growth. However, when β-glucosidases encapsulated and transported by bacterial MVs are provided, the receivers are able to grow on polysaccharides, allowing for the production of PBPs and the simultaneous spatial separation of the receptor from the producer, promoting growth (43) (**Figure 3**). This modus operandi allows the receiver to conditionally surpass the producer, indicating significant potential utility and value in bacterial MVs and their content components in the realm of bacterial nutrient acquisition.

### 2.2 Inter-cell communication

**Figure 4:**
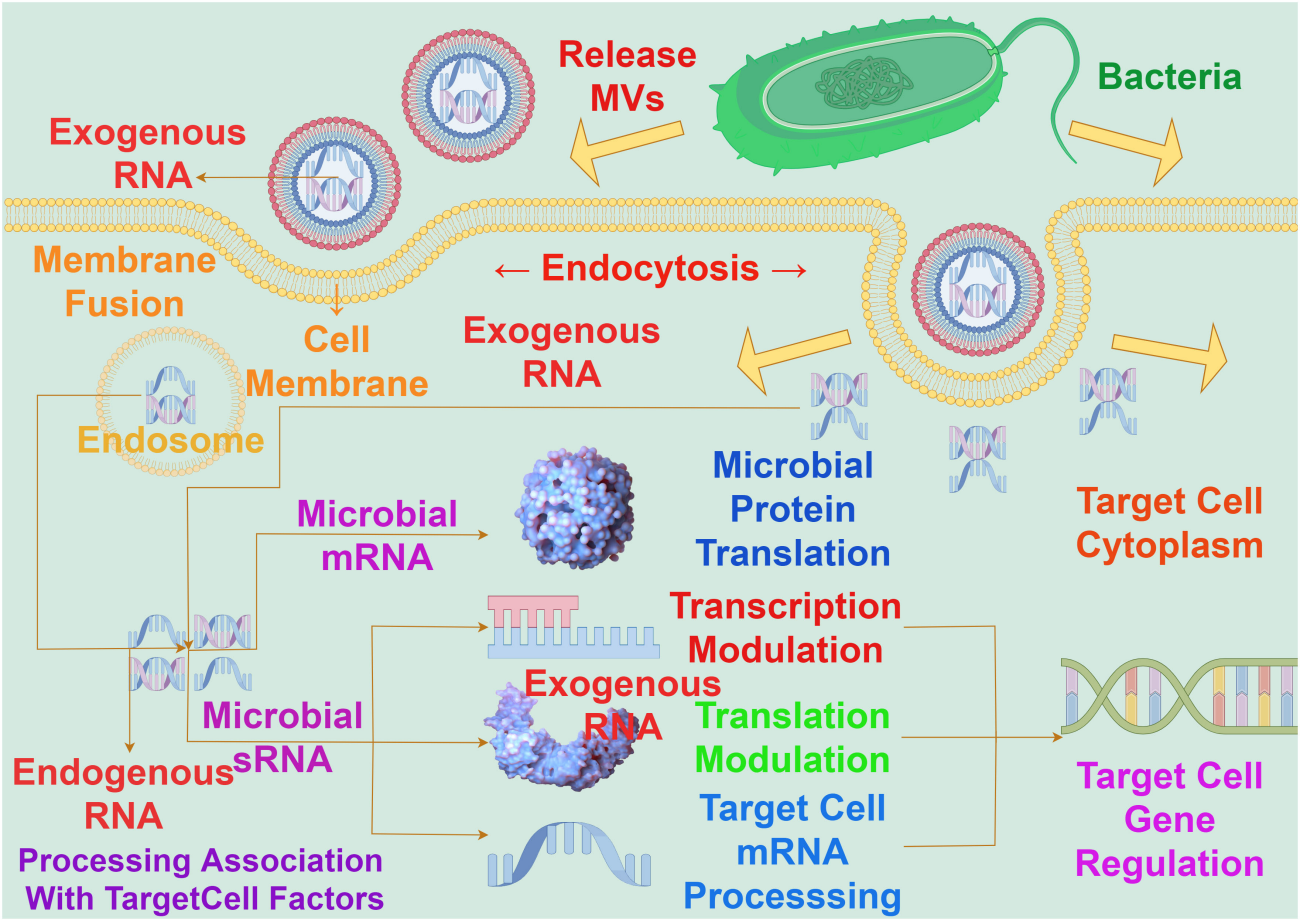
The interaction between bacterial MVs and RNA on gene expression in target cells. The RNA carried by MVs must enter the cytoplasm to exert an influence on gene expression in target cells, a process achieved through fusion with the target cell membrane. The concerted action of exogenous RNA carried by MVs and endogenous RNA within the nucleus involves interactions with target cell factors. This interaction operates at the levels of messenger RNA and sRNA, impacting transcriptional regulation, translational regulation, target cell mRNA processing, and microbial protein translation, so as to realize the process of exogenous RNA regulating host gene expression.

Bacterial MVs play a critical intermediary role in the communication among cells. These MVs contain functional components such as proteins, lipids, and nucleic acids, allowing them to transfer signaling molecules among cells, thereby influencing the behavior and physiological state of bacterial communities. Bacterial MVs can act as messengers between bacterial cells, transmitting information through carrying specific bioactive molecules such as genetic material, metabolites, and signaling molecules. The protective membrane structure of MVs maintains the stability of these molecules, allowing them to influence the activities and responses between bacteria and cells through long-distance transmission. For example, studies have observed that in the process of molecular transfer in *Acinetobacter baylyi*, MVs containing DNA first rupture upon contact with the bacteria’s outer membrane, followed by DNA import mediated by type IV pili (44).Bacterial MVs have the capacity to transport RNA into eukaryotic cells. The transported RNA within bacterial MVs is delivered to the host cell, by membrane fusion with the host cell membrane, to modulate gene expression by targeting and regulating the translation or stability of host cell mRNA (**Figure 4**). It has been demonstrated that exogenous sRNAs detected in periodontal pathogens MVs can inhibit the expression of certain cytokines in Jurkat T cells (45), and these MVs’ sRNAs can identify potential target genes related to human immunity (46).

Different vesicles produced by bacterial strains typically contain common substances but also exhibit significant strain-specific differences. These differences are reflected in the varied contents of bacterial MVs due to the growth stages of the strain. Analysis has revealed substantial differences in the protein composition of *Helicobacter pylori* MVs during bacterial growth, with MVs containing a greater variety of proteins compared to their parent (47). The expression levels of IL-8 also exhibit significant variations throughout bacterial growth, with a noticeable increase in immunogenicity across the entire growth process (48). Consequently, the size, protein composition, and immunogenicity of MVs generated at different growth stages are incomparable. Overall, these findings underscore the importance of considering the bacterial growth stage in the isolation of MVs. As a major autolysin of *P. aeruginosa* PAO1, the protease is associated with the natural release of MVs from the cell surface during bacterial growth. Its expression is linked to the growth stage of the bacteria; in batch cultures, it is predominantly detected in the mid-exponential growth phase, whereas in synchronized cultures, it is mainly detectable during cell elongation and division phases (49). Consequently, the growth stage of the strain can influence the size, protein composition, and cargo packaging of its MVs. This heterogeneity of content causes MVs formed at different growth stages to transmit varied information to recipients, resulting in diverse regulatory effects.

Numerous bacteria utilize extracellular signaling molecules to coordinate group behaviors through a process termed quorum sensing (QS). However, some hydrophobic QS molecules are unable to diffuse across the bacterial outer membrane. Studies have shown that *Vibrio harveyi* sequesters the hydrophobic QS molecule CAI-1 long chain ketone into bacterial MVs, releasing it into the environment. This enables the stable distribution of CAI-1 in aqueous environments over long distances through the assistance of MVs (50). Similarly, *Paracoccus denitrificans* packages the hydrophobic molecule C16-HSL into MVs, facilitating its release and transfer in water environments to other bacteria with distinct tendencies (51). It has been demonstrated that in the absence of MVs, inter-bacterial communication within bacterial communities is likely to be suppressed. For instance, *P. aeruginosa* utilizes MVs to package and transfer signaling molecules such as the Pseudomonas quinolone signal (PQS) for intracommunity communication (18). PQS stimulates MV production by inserting into the outer membrane (19), and the concentration of PQS in MVs is high. A minimal fusion of MVs with bacterial cells is sufficient to trigger quorum sensing. Hydrophobic signaling molecules associated with quorum sensing serve as cargo transport in MVs, a process commonly observed in other microorganisms (50). Removal of these MVs from the bacterial community disrupts intercellular communication and suppresses PQS-controlled group behaviors. This underscores the significance of vesicle cargo contents in microbial populations and emphasizes the crucial role of MVs in mediating intercellular communication among bacterial cells.

### 2.3 Environmental adaptation

Bacterial MVs play a vital role in environmental stress response. When bacteria are exposed to environmental pressures such as changes in temperature, pH, or oxygen concentration, they produce a higher abundance and different types of MVs. This may be a bacterial adaptation and regulatory strategy to cope with environmental changes. Under environmental stress conditions, the production and cargo of functional molecules in bacterial MVs significantly change, thereby modulating bacterial physiological status and adaptability. For instance, *Staphylococcus aureus* exhibits higher MV production under oxidative stress, iron-limited conditions, or sub-inhibitory concentrations of ethanol. Conversely, hyperosmotic stress or sub-inhibitory concentrations of erythromycin significantly reduce the production of *S. aureus* MVs (52). *P. aeruginosa* can increase the production of MVs when exposed to certain environmental stressors, while also regulating the activity of the periplasmic protease MucD expression factor AlgU. Although overexpression of AlgU is sufficient to induce MVs production, strains lacking AlgU still exhibit increased MV generation under stress conditions, suggesting that stress-induced MVs production does not depend on the activation of AlgU (53). The generation of vesicles occurs in infected tissues and is easily influenced by environmental factors (54), typically driven by phospholipid accumulation in the outer membrane and regulated by the phospholipid transporter protein VacJ/Yrb. In *Haemophilus influenzae* and *V. cholerae*, the absence or inhibition of VacJ/Yrb can increase MVs production, leading to the enrichment of phospholipids and certain fatty acids in these MVs, indicating that MV production is related to the regulation of the VacJ/Yrb ABC transport system and iron-deficiency status (16). Studies have demonstrated that VacJ/Yrb is suppressed in the early stages of mammalian infection, which stimulates the rapid formation of MVs to facilitate bacterial surface exchange and adaptation to the host environment (55).

Bacteria can also utilize MVs to exchange cell surface components, enabling them to rapidly sense and respond to environments with nutrient deficiencies. For example, the *Pseudomonas putida* DOT-T1E strain releases MVs within ten minutes after exposure to stressors, leading to a significant increase in the hydrophobicity of the cell surface (56). This assists in regulating bacterial adaptation to the host environment. Such surface changes can also facilitate effective processing of carbohydrates, reducing the loss of substrates and enzymes due to diffusion (33). Furthermore, *Vibrio fischeri*, when grown under acidic pH conditions, upregulates the transcription of its major outer membrane protein OmpU, which is controlled in MVs based on nutritional conditions. Upon encountering acidic pH in the host, the MVs of *Vibrio fischeri* serve as effective stimulatory factors for symbiotic host development in an OmpU-dependent manner (57). In addition, the psychrophilic Gram-negative bacterium *Pseudoalteromonas antarctica* NF3, isolated from Antarctica, can amass substantial quantities of high-protein extracellular polymeric substances (EPS), comprising capsule polymers and abundant MVs with the capacity to transport proteins into the external environment. This characteristic plays a crucial role in the bacterium’s survival under the extreme low-temperature conditions prevalent in its habitat (58). Significantly, these studies underscore the essential role of MVs in bacterial adaptation to extreme conditions.

### 2.4 Antibiotic correlation

Numerous investigations have substantiated the integral role of extracellular vesicles (MVs) in modulating bacterial physiological processes, particularly in the context of antibiotics. This involvement manifests predominantly in two facets: shielding bacteria from antibiotic-induced damage and utilizing MVs as vehicles for antibiotic secretion, thereby contributing to inter-bacterial competitive dynamics. Bacterial MVs play a critical role in mediating the resistance of bacterial communities to antibiotics. For example, MVs of the Antarctic bacterium *Pseudomonas fluorescens* offer protection to the producer from the effects of membrane-active antibiotics, such as colistin and melittin, with their membrane and protein packaging closely resembling those of the parent bacteria (59). The protective mechanism employed by MVs shields bacteria from the impact of membrane-active antibiotics, albeit ineffective against erythromycin. MVs can reduce the susceptibility of bacteria to antibiotics by secreting antibiotic-degrading enzymes or by regulating the permeability of bacterial membranes, thus providing bacteria with a certain level of resistance to antibiotics. studies have shown that bacterial MVs of *E. coli* can secrete membrane-anchored β-lactamase (NDM-1) (60). Membrane anchoring facilitates the secretion of NDM-1 enzyme in bacterial MVs and enhances its stability. MVs containing NDM-1 can provide protection to nearby bacterial populations, shielding them from the effects of other lethal antibiotics. The complex structure and composition of MVs enable them to modulate their action on bacteria by adsorbing, encapsulating, or binding antibiotics. However, the protective effects of bacterial MVs are not universal for all antibiotics, such as ciprofloxacin, erythromycin, and trimethoprim, among others. This suggests that MVs may only protect bacteria from antibiotics targeting the bacterial membrane. This process is likely achieved through membrane encapsulation and sequestration of antibiotics, thereby reducing the damage inflicted by antibiotics on bacteria (61).

**Figure 5:**
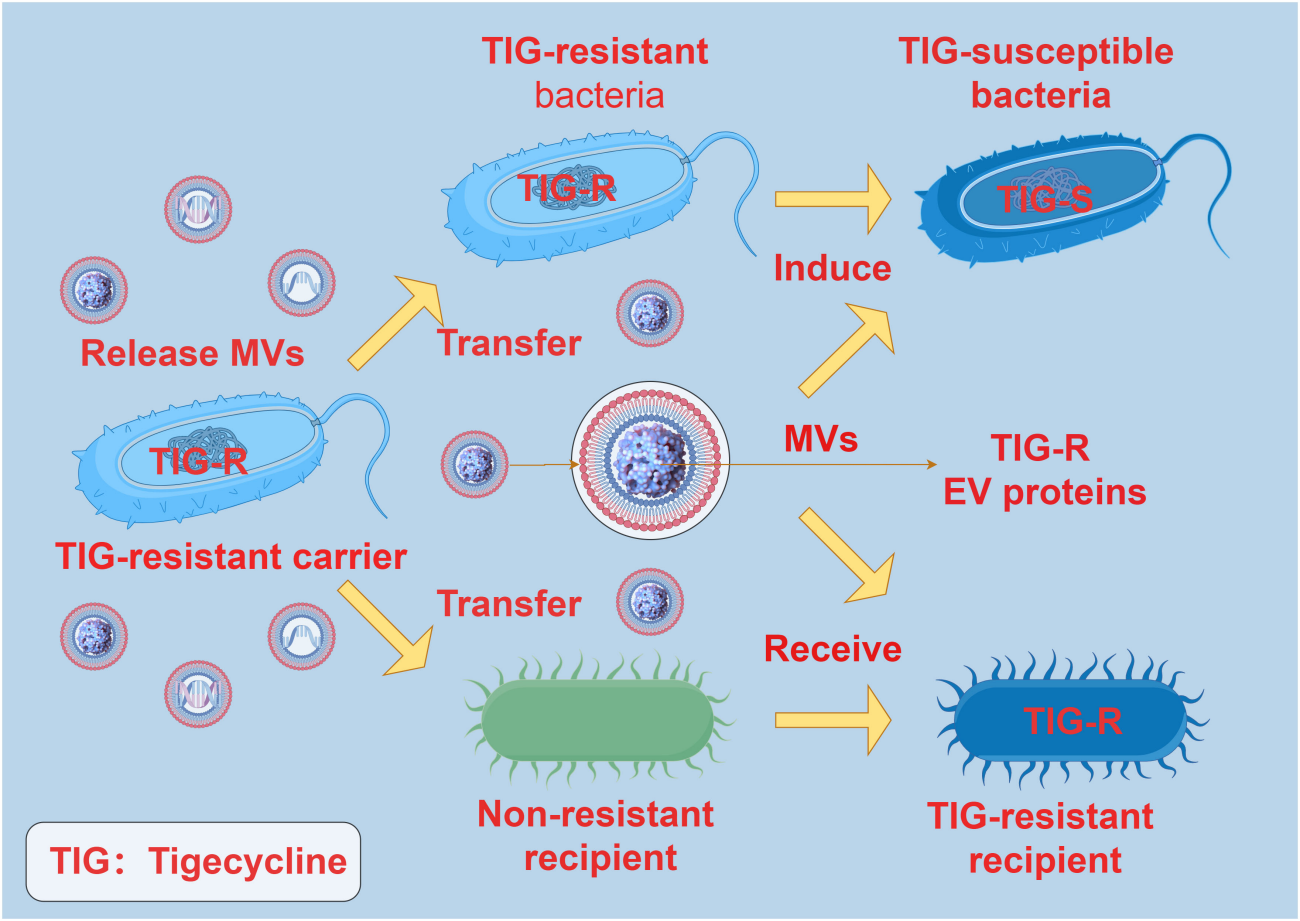
The interaction between MVs and antibiotic resistance. TIG-R EV protein is the main factor of antibiotic resistance transfer of TIG. The TIG-R EV protein carried by the MVs of the TIG-resistant carrier can be selectively transferred to the non-resistant recipient, and the non-resistant recipient produces TIG resistance after receiving the TIG-R EV protein carried by the MVs. TIG-resistant bacteria became TIG-susceptible bacteria under the induction of MVs carrying TIG-R EV protein.

When bacteria are challenged with low doses of antibiotics, the production and transportation of MVs are significantly enhanced. This enhanced vesicle movement is correlated with a decrease in the density of surface appendages responsive to antibiotics, independently of cell aggregation characteristics (62). Bacterial MVs can interact with antibiotics through specific receptors on the membrane, thereby reducing the antibiotic concentration and easing antibiotic pressure on the bacteria (63). Research indicates that following short-term exposure to antibiotics, the quantity of bacterial MVs increases, suggesting potential sequestration of antibiotics within the MVs. Subsequent investigations revealed that following co-incubation of MVs with animal cells, antibiotics were detectable within the cytoplasm of the animal cells (64). Interestingly, some studies have also found that there is antibiotic resistance transfer via MVs between bacteria. In *Acinetobacter baumannii*, the TIG-R EV protein carried by MVs plays a pivotal role in the transfer of tigecycline (TIG) resistance. Outer membrane vesicles selectively transfer TIG resistance to neighboring bacterial cells, resulting in neighboring bacteria developing highly selective TIG resistance, with MVs being more likely to induce TIG resistance compared to antibiotics (65) (**Figure 5**). These findings are essential for comprehending the role of MVs in the development and transmission of antibiotic resistance in diverse bacterial strains. This not only suggests that released bacterial MVs can serve as a mechanism for extruding antibiotics, but also as a means for transmembrane transport, allowing antibiotics that are typically impermeable to cell membranes to be delivered into cells, thereby exerting their effects across cell membranes.

The role of MVs is also reflected in participating in the competition among bacteria. It has been demonstrated that bacteria are capable of secreting MVs carrying antimicrobial components. For example, *Chromobacterium violaceum* utilizes bacterial MVs to solubilize and transport violacein to other microorganisms (66). Pyocyanin, as an active antibiotic component within MVs, is a hydrophobic indole with known antibacterial activity against other microorganisms. By carrying antibiotics, MVs inhibit the growth of other microbial communities in the environment, granting the bacterial population a competitive edge and enhancing the inter-species competitive ability of specific microbial populations. Additionally, MVs of *Burkholderia thailandensis* contain hydroxy-alkyl quinolines (HAQs) and long-chain rhamnolipids, among other antimicrobial compounds. These compounds exhibit antimicrobial and anti-biofilm properties, capable of inhibiting the growth of drug-resistant bacteria and fungi (67). Some bacterial MVs also participate in bacterial antibiotic resistance regulation through active substances such as hydrolytic enzymes. For example, *P. aeruginosa* can secrete two types of MVs containing peptidoglycan hydrolase (autolysin), the expression of which is growth-phase dependent. These enzymes can hydrolyze and separate both Gram-positive and Gram-negative cell capsules, as well as several types of glycine peptides. Research has shown that n-MVs can kill *P. aeruginosa* cultures with permeability-resistant to gentamicin, indicating fusion of n-MVs with the outer membrane, releasing autolysin into the periplasm, where they degrade peptidoglycan and dissolve the cells. On the other hand, g-MVs release gentamicin and autolysin into resistant cells, inhibiting the growth of various strains including opportunistic pathogens, Gram-positive and Gram-negative bacteria. This inhibitory effect is caused by certain substances within the MVs, rather than just physical contact (68). In summary, bacterial MVs is closely related to antibiotics in the regulation of bacterial physiological activities and plays a variety of functions in the interaction between bacteria and antibiotics. These findings provide new insights for the study of antibiotic resistance and the application of MVs in antibiotic treatment.

## 3 Function of bacterial MVs in pathogenic process

Bacterial MVs have emerged as essential mediators in a variety of pathogenic mechanisms, including the release of virulence factors, invasion of host cells, formation of biofilms, and modulation of host immune responses. Understanding the multifaceted functions of bacterial MVs in pathogenesis is of paramount importance in deciphering pathogenicity, host–pathogen interactions, and the development of novel therapeutic strategies. Thus, elucidating the intricate roles of MVs in bacterial pathogenicity will undoubtedly provide valuable insights for both fundamental research and clinical applications.

### 3.1 Release virulence factor

During the pathogenic infection process in bacteria, bacterial MVs can transfer virulence factors, such as toxins, adhesins, degradative enzymes, and effector molecules, to host cells, encapsulating these factors within the MVs. For example, gingival protease serves as the primary virulence factor in *P. gingivalis*, leading to the impairment of host cell function (14). *Vibrio tasmaniensis* LGP32’s virulence factor, the caseinase/gelatinase (Vsp), is specifically secreted through enclosed MVs, participating in delivering the virulent phenotype to host immune cells and providing protection against antimicrobial peptides (69). MVs have a highly protective effect on their contents, ensuring their protection from degradation. For instance, *V. cholerae*’s MVs can effectively deliver biologically active cholera toxin to intestinal epithelial cells, protecting the toxin from degradation by intestinal proteases and delivering active cholera toxin to host cells (70). This indicates that the protection provided by MVs allows these toxins to remain active in the intestine for a longer period, increasing their stability and prolonging their activity.

MVs are typically released into the immediate environment of bacteria or into host cells to exert their effects. For example, it has been confirmed that all C-terminal domain (CTD) proteins and other virulence factors are enriched in MVs, demonstrating that *P. gingivalis* can selectively concentrate numerous virulence factors within MVs and release them into the environment (71). The secretion of virulence factors by bacteria does not involve the release of naked proteins into the surrounding environment. Instead, it relies on the interaction of specific substances or receptors on MVs or host cell surfaces, selectively targeting the delivery of virulence factors to host cells. For instance, the cholesterol-dependent fusion of *Staphylococcus aureus* MVs with the host cell membrane is a pathway for the targeted delivery of the key virulence factor alpha-toxin to human cells (72). α-Toxin is a protein associated with MVs responsible for red blood cell lysis and is closely related to MVs isolated from the *Staphylococcus aureus* strain 8325-4. Enterotoxigenic *E. coli* (ETEC strains), which produce enterotoxins, are commonly associated with diarrheal diseases. Acting as specific targeted transporters, MVs mediate the entry of active enterotoxins and other bacterial envelope components into host cells (73). Several secreted toxins, including heat-labile enterotoxin and cytotoxic protein cytolysin A (ClyA), have been detected in their MVs (74, 75). Additionally, their MVs contain a biologically active substance capable of calcium-dependent binding to red blood cells, forming a biologically potent form of RTX toxin associated with MVs, thereby enhancing their potential against target cells (76). *P. aeruginosa* secretes MVs that, through fusion with lipid rafts in the host membrane, directly transport various virulence factors (including β-lactamase, alkaline phosphatase, hemolytic phospholipase C, and Cif) into the host cell cytoplasm via N-WASP-mediated actin transport. These factors rapidly distribute within the host to specific target cell locations, exerting their effects on the host (77). This suggests a possible mechanism for the release of virulence factors by *P. aeruginosa*, Legionella pneumophila, and Streptomyces through MVs (77–79).

In the course of bacterial pathogenic infections, MVs serve as carriers to transport a diverse and intricate set of virulence factors, exerting a multitude of functions that can directly or indirectly modulate various complex physiological processes within host cells. For example, protein group analysis of *M. tuberculosis* MVs revealed the presence of various virulence factors, including FadA, MORN2, and YadA (80). *Acinetobacter baumannii* exploits MVs, which contain outer membrane proteins, to enhance bacterial pathogenesis and dissemination. Specifically, MVs carrying OmpA, upon internalization by host cells, trigger activation of the host GTPase dynamin-related protein 1 (DRP1). This OmpA-induced activation of DRP1 leads to increased accumulation on mitochondria, resulting in mitochondrial fragmentation, heightened reactive oxygen species (ROS) production, and ultimately, cell death (81). Furthermore, MVs from *P. gingivalis* carrying proteins with OmpA peptidoglycan-binding motifs and TonB-dependent receptors preferentially adhere to the cell membrane surface. These findings underscore the diverse and profound impact of MVs on bacterial pathogenesis and host interactions (71). During the infection process, *Yersinia pestis* releases membrane vesicles (MVs) which increase in response to membrane stress and mutations in RseA, Hfq, and the major Braun lipoprotein (Lpp). These MVs contain the catalytically active Pla, which promotes plasminogen activation and α2-antiplasmin degradation (82). Moreover, MVs can bind to extracellular matrix components such as fibronectin and laminin, and it is hypothesized that the diffusion process of Pla through MVs may influence infection outcomes through interactions with Pla substrates such as plasminogen and Fas ligand. These findings highlight the important role of bacterial MVs in the delivery of virulence factors and provide a new perspective for understanding the mechanisms of pathogenic microbe-host interactions.

### 3.2 Host cell invasion

Bacterial secreted MVs contain a variety of factors that can assist in bacterial invasion of the host and participate in the infection process, serving as carriers that enter host cells to exert their effects. For example, the MVs of *Edwardsiella piscicida*, acting as carriers for hemolysins, can induce caspase-dependent apoptotic-like cell death in fish non-phagocytic cells during infection, ultimately inducing intestinal inflammation to promote bacterial colonization within the host (83). The hemolysin primarily binds to bacterial MVs and is internalized into fish cells through dynamin-dependent endocytosis, subsequently inducing pyroptotic cell death. Additionally, MVs secreted by *Pseudoalteromonas* sp. strain KS62 carry active K-carrageenase, which can successfully degrade the main polysaccharides of red algae cell walls such as kappa-carrageenan, enabling bacteria to invade and colonize algal hosts and thereby establish a saprophytic mode of life (35). The MVs harbor invasive protein antigens, such as IpaB, IpaC, and IpaD, which have been identified in *Acinetobacter baumannii* MVs (65). Their double-layered surface is comprised of LPS and Ipa proteins, which are instrumental in facilitating MVs’ invasion of host cells through membrane fusion. These MVs are readily internalized by non-phagocytic membranes and subsequently deliver their contents into the cytoplasm of host tissues. Additionally, MVs released by *B. subtilis* have been shown to transfer the virus receptor YueB through membrane fusion, thereby enhancing the invasion and adsorption of the bacteriophage SPP1 onto non-host species (84). Studies have demonstrated that this molecular exchange is mediated by MVs, a phenomenon known as Acquiring Sensitivity to Exogenous DNA (ASEN). MVs secreted by the Antarctic halophilic archaeon *Halorubrum lacusprofundi* are capable of infecting plasmid-free strains and subsequently acquiring the capacity to produce vesicles containing plasmids (85). Moreover, proteins encoded by the archaeal plasmid pR1SE have been detected within regularly-shaped membrane vesicles that encapsulate plasmid DNA, potentially integrating and replicating as part of the host genome. These plasmids bind to host DNA fragments or undergo partial degradation to form MVs, ultimately transferring to new hosts.

**Figure 6:**
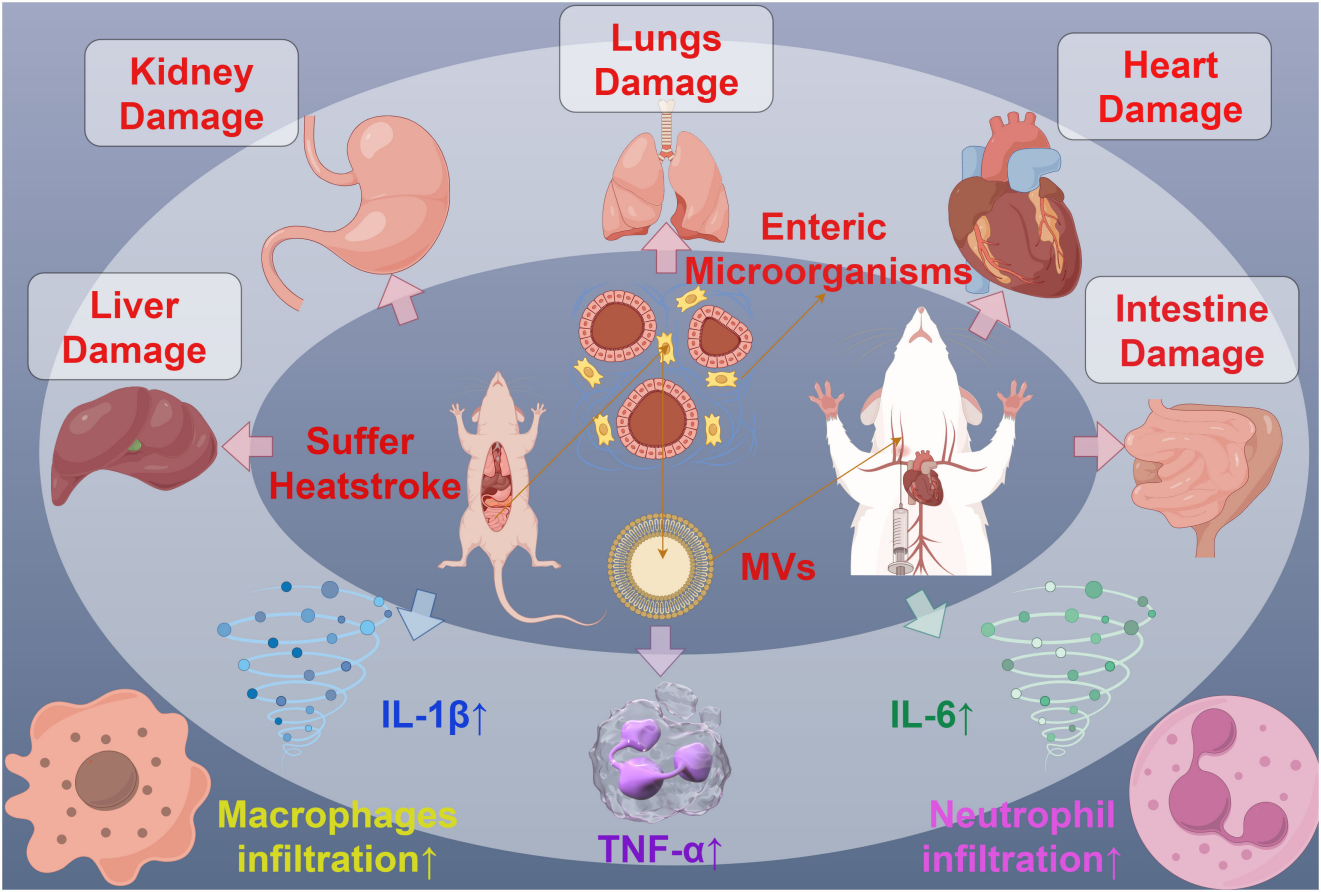
The interaction between MVs and organ injury in mice. MVs derived from mouse Intestine microorganisms of Suffer Heatstroke, after injected into mice, caused pathological changes of multiple organs, including the damage of liver, kidney, lungs, heart and intent, increased inflammatory cell infiltration and increased inflammatory cytokines such as IL-1β, IL-6 and TNF-α.

MVs can also interact with specific host cells through the substances or receptors on their membrane surface, participating in the process of host invasion. For example, MVs from oligodendrocytes have been found to play a functional role in the transport of endocannabinoids. The MVs’ surface carries N-acylethanolamine (N-AEA), which can stimulate the type 1 cannabinoid receptor (CB1) and inhibit presynaptic transmission to GABAergic neurons (86). Furthermore, symbiotic gut bacteria have evolved to transfer secreted effector molecules in the form of MVs, selectively targeting parasites. Under strong induction by host serum, the *Serratia marcescens* Su_YN1 releases MVs and the anti-malarial protein AmLip into the mosquito gut (87). AmLip is first secreted through T1SS into the extracellular space, then preferentially attaches to MVs selectively targeting malaria parasites, ultimately invading and killing them. Research has shown that the invasion of MVs significantly affects the induction of organ pathological changes, inflammatory infiltration, the expression of inflammatory cytokines, and serum organ damage biomarkers. During severe heatstroke, MVs can induce acute organ damage, with a significant increase in the MVs produced by the gut microbiota of heatstroke mice. These MVs massively invade various organs in the mice, particularly accumulating significantly in the liver and lungs, leading to significant organ pathology changes, increased infiltration of inflammatory cells (macrophages and neutrophils), expression of inflammatory cytokines (TNF-α, IL-1β, IL-6), and serum biomarkers of organ damage. Conversely, inhibiting endogenous MVs can alleviate the organ damage caused by heatstroke (88) (**Figure 6**). Bacterial MVs can also achieve cell-specific targeting and kill cancer cells by releasing small interfering RNA (siRNA) targeted at tubulin-associated proteins. Using modified *E. coli* strains with reduced endotoxin effects on human cells, MVs displaying human epidermal growth factor receptor 2 (HER2)-specific core-structured proteins were produced as targeting ligands in their membrane. These ligands, delivered through systemically injected MV-packaged siRNA, resulted in targeted gene silencing, successfully invading tumor cells in animal models, leading to a significant reversal of tumor growth (89). These findings reveal the complex role of bacterial MVs in regulating the infection process, indicating the potential value of MVs in the invasion of host cells and providing a novel perspective for understanding the pathogenic mechanisms of microorganisms.

### 3.3 Biofilm formation

Bacteria can thrive in natural environments through their ability to grow in an attached manner. The formation of a biofilm - a community of bacterial cells enveloped in a self-produced extracellular matrix - serves to protect the cells from external factors such as antibiotics and host immune responses. Bacterial MVs can provide essential components required to build the biofilm by carrying bioactive molecules such as proteins, phospholipids, and polysaccharides, directly contributing to the formation and modification of bacterial biofilms. Biofilms are well-structured communities, and their formation process includes initial bacterial adhesion, biofilm development, and mature diffusion. Bacterial MVs can serve as scaffolds for bacterial adhesion and aggregation and can deliver key molecules such as bacterial surface structure proteins and outer membrane vesicles to the external surface of bacterial cells. Early studies have shown that specific proteins in the MVs released by the *Helicobacter pylori* strain TK1402 can participate in bacterial aggregation and biofilm formation, and the addition of these MVs can significantly increase the formation of the biofilm in a dose-dependent manner (90). Furthermore, a similar trend has been observed in *P. aeruginosa* strains. The components of the biofilm formed by *P. aeruginosa* strains include polysaccharides containing GlcNAc, extracellular nucleic acids, and proteins. Experimental evidence has shown that the amount of biofilm formation increases with the addition of purified MVs, particularly in the case of an increase in the amounts of eDNAs and GlcNAc, which are believed to constitute the polysaccharides of the biofilm, following the addition of purified MVs (91). This suggests that the formation of biofilms in *P. aeruginosa* strains is significantly influenced by the release of MVs. The secretion of MVs by bacteria is closely related to the physiological status and environmental stimuli affecting biofilm formation. The generation of MVs has been established as an independent bacterial stress response pathway. When bacteria encounter environmental stress, such as changes in the environment experienced during the colonization of host tissues, this pathway may be activated (92). For instance, in conditions where the toxic levels of long-chain alcohols are present, induced osmotic pressure changes brought about by NaCl, the presence of EDTA, and subsequent heat shock, cells of the *P. aeruginosa* strain DOT-T1E release MVs within ten minutes under stress conditions, leading to a significantly enhanced biofilm formation in this scenario (56). In the initial stages of biofilm formation, the extracellular matrix plays a crucial role in bacterial adhesion to surfaces and cell-to-cell interactions (93). Studies have indicated that MVs are only detected within the matrix of the biofilm formed by *Helicobacter pylori* strain TK1402 (90), suggesting that MVs play a significant role in the formation of the extracellular matrix in the biofilm of this bacterial strain. The biofilm matrix serves as the chemical, structural, and functional foundation of the biofilm, and matrix components derived from biofilm such as MVs and DNA exhibit concentration, pH, and cation-dependent interactions (94). The binding of MVs to DNA impacts the surface properties of MVs, thus influencing the reactivity and efficacy of the interactions of the matrix polymers and other components.

**Figure 7:**
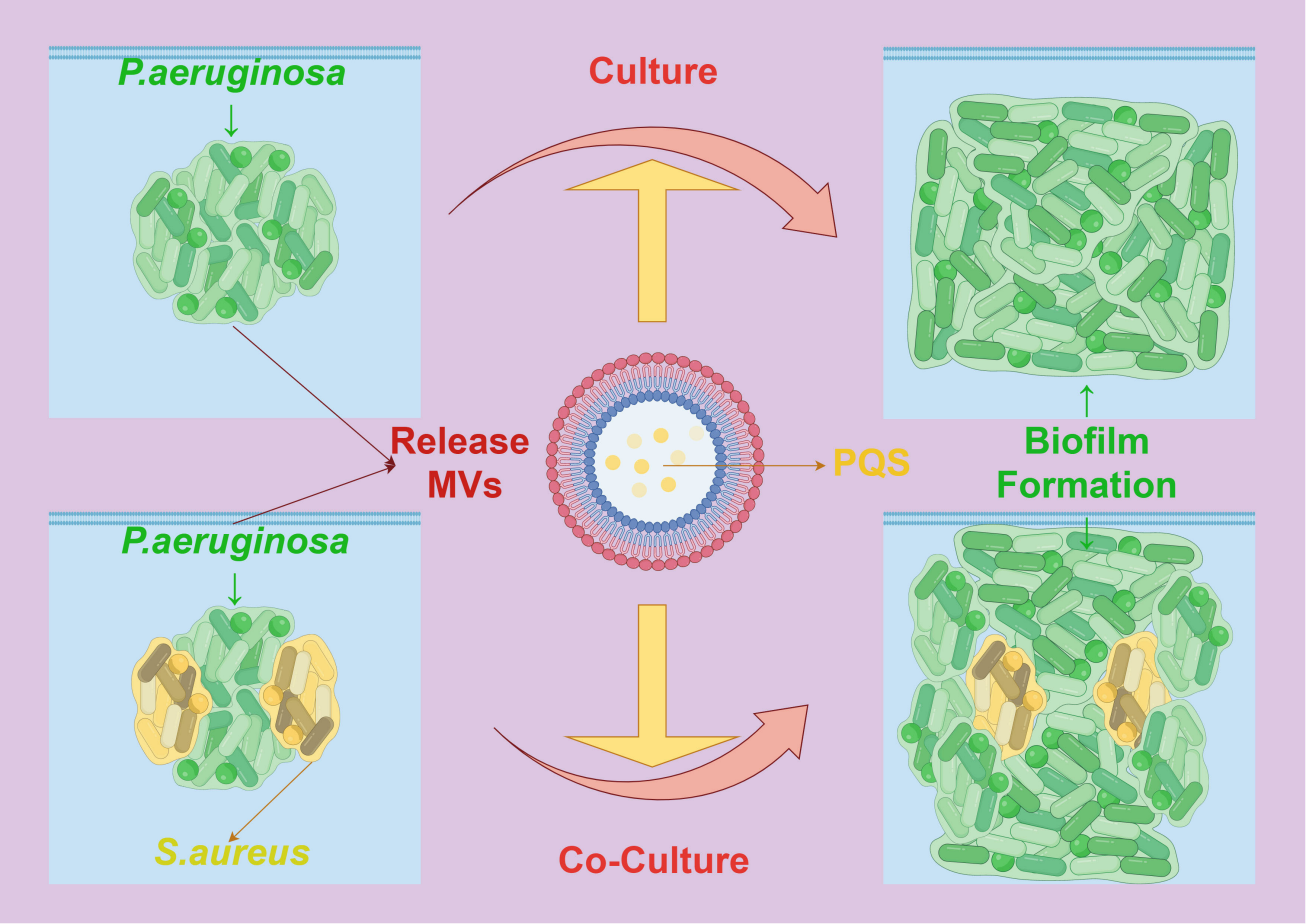
The interaction between MVs and biofilm formation. PQS mediated by *Pseudomonas aeruginosa* MVs causes the cells in the biofilm to be stretched, deformed and contacted with the surrounding cells, which promotes the formation of its own biofilm. In the composite biofilm formed by *Pseudomonas aeruginosa* and *Staphylococcus aureus*, the mediation of MVs enhanced the inhibitory effect of PQS on the growth and biofilm formation of *Staphylococcus aureus*.

Additionally, research has shown that MVs secreted by certain bacteria can inhibit the biofilm formation of other bacteria, displaying a competitive mechanism. For instance, MVs produced by *P. aeruginosa* contain a quorum-sensing signal, the *Pseudomonas* quinolone signal (PQS), which significantly inhibits the biofilm formation of *Streptococcus pneumoniae*, without affecting its cell growth (95). In some bacteria, MVs also have a function in regulating intracellular and extracellular enzymes and biofilm assembly. Under the mediation of MVs, the structure and composition of extracellular polymeric substances in *P. aeruginosa* undergo significant changes due to PQS regulation mediated by MVs. MV-mediated PQS promotes the growth of the biofilm, causing cells in the biofilm to elongate, deform, and make contact with surrounding cells in a bridging manner, with extracellular proteins rather than polysaccharides playing a primary role in this process. In composite biofilms formed by *P. aeruginosa* and *Staphylococcus aureus*, the mediating role of MVs enhances the inhibitory effect of PQS on the growth of *S. aureus*, resulting in reduced production of extracellular polysaccharides by both bacteria (96) (**Figure 7**). These changes also lead to variations in the richness, diversity, and structure of microbial communities in biofilms formed by activated sludge. Therefore, MVs provide components of the extracellular matrix and signaling molecules that promote biofilm formation and maturation in the process of bacterial biofilm formation, playing a crucial role in mediating interactions between bacterial cells and demonstrating their key function in the formation of bacterial biofilms.

### 3.4 Immunoregulation

MVs are rich in various bioactive molecules, that regulate host immune responses. For instance, the MVs of *P. aeruginosa* PA14 contain abundant sRNA that is differentially packaged and can dampen host immune responses. Specifically, the sRNA52320 significantly reduced the LPS-induced and MVs-induced IL-8 secretion in primary human airway epithelial cells after transfer, and also markedly attenuated the MVs-induced KC cytokine secretion and neutrophil infiltration in mouse lungs (97). Furthermore, MVs can spread through blood flow and the lymphatic system to other tissues and organs, interacting with the host’s immune cells such as lymphocytes and macrophages, and regulating the host’s immune response, cell activation, and inflammatory reaction. The MVs of *Burkholderia cepacia* ATCC 7966TM can induce the overexpression of proinflammatory cytokines, promoting the activation and apoptosis of mononuclear lymphocytes (98). On the other hand, MVs from *Fusobacterium nucleatum* can polarize macrophages toward an M1-like phenotype in a murine model of periodontal disease, promoting the development of periodontitis (99). During its growth, pathogenic bacteria continuously release MVs containing toxic bacterial components, which regulate the degree of inflammatory response by controlling the functions of immune and non-immune cells in the tissue (100). MVs derived from Helicobacter pylori carries virulence factors such as cytotoxin-related gene A (CagA) and vacuolated cytotoxin A (VacA), and it has been proved that gastric administration of MVs from Helicobacter pylori can promote atherosclerosis by inducing pro-inflammatory reaction and plaque formation (101).

Furthermore, *Neisseria gonorrhoeae* depends on the bacterial outer membrane porin protein (PorB) to evade innate immunity and colonize the mucosa of the reproductive tract. This process involves *Neisseria gonorrhoeae* using MVs to target PorB to the host mitochondria, promoting infection and modulating the cell apoptosis pathway to induce macrophage apoptosis, thus affecting innate immunity (102). Research also confirms that macrophage responses to the protein composition of MVs require adjuvant-like activity from LPS to trigger strain-specific responses (103). Protease treatment that removes proteins from the vesicle surface leads to reduced production of interleukin-6 and tumor necrosis factor-α, indicating that the production of these specific cytokines is related to macrophage recognition of MV-associated proteins.

Research has shown that MVs can induce innate immune responses in cells and promote the production of pro-inflammatory cytokines, thereby enhancing the immune response. For example, the MVs of *Klebsiella pneumoniae* ATCC 13883 are capable of inducing the expression of pro-inflammatory cytokine genes such as interleukin (IL)-1β and IL-8 in epithelial cells during *in vitro* cultivation, without inhibiting cell growth or inducing cell death (104). MVs can also influence the signal transduction pathways and immune responses of recipient cells, affecting their function and expression. Furthermore, MVs from *P. gingivalis* can induce strong TLR2 and TLR4-specific responses as well as moderate responses in recipient cells, reflecting their action on different pattern recognition receptor responses (105). MVs from *Burkholderia cenocepacia* stimulate the production of IL-8 and TNF, and their lipopolysaccharides can further activate immune cells to trigger an inflammatory response by binding to immune receptors such as Toll-like receptor-4 (TLR-4) (106). It has been demonstrated that bacterial MVs have a significant ability to effectively induce long-term anti-tumor immune responses. Modified MVs from *E. coli* W3110 can specifically target accumulation in tumor tissues, subsequently inducing the production of the anti-tumor cytokines CXCL10 and interferon-γ, completely eradicating established tumors without significant adverse reactions. This anti-tumor effect depends on interferon-γ, as interferon-γ-deficient mice do not induce this MV-mediated immune response (107). These findings demonstrate the potential value of MVs in modulating the immune regulation process through their transport to recipient cells and targeted regulation of various physiological processes. Therefore, the study of the mechanisms underlying MV regulation in immunomodulation provides new insights for the development of novel MV-based immunotherapeutic strategies.

## 4 Methods for bacterial MVs extraction and analysis

The isolation, quantification, and characterization of bacterial MVs are essential for understanding their role in environmental adaptation and pathogenic processes. Various methods have been established for the extraction and analysis of bacterial MVs, each with its advantages and limitations.

Ultracentrifugation stands as the most widely used method for isolating bacterial MVs. By subjecting bacterial culture supernatant or vesicle-rich fractions to high-speed centrifugation, MVs are precipitated. However, ultracentrifugation does not completely separate membrane fragments from protein aggregates. To address this issue, a combination of density gradient with ultracentrifugation is commonly employed for more effective MV extraction (21, 108, 109). The filtration method involves the use of filters with varying pore sizes to separate bacterial MVs from the cells based on their size and charge. Typically, the upper layer of the bacterial culture is filtered through membranes with pore sizes ranging from 0.22 to 0.45 μm to extract MVs. Subsequently, the bacterial supernatant is passed through a membrane with a specific molecular weight cutoff, typically 50–100 kDa, to remove most of the proteins unrelated to MVs (110, 111). The precipitation method involves the addition of various salts, polymers, or solvents to the bacterial culture supernatant or vesicle-rich fractions to induce the precipitation of bacterial MVs, providing an alternative to the ultracentrifugation and filtration methods. Electron microscopy is utilized to observe the morphology and structure of bacterial MVs, and MVs labeled with fluorescent dyes used to study its biological distribution (73), facilitating analysis of their size distribution and cargo composition at a visual level. Proteomics and Lipidomics methods provide insights into the biogenesis and composition classification of bacterial MVs by describing their protein and lipid content, shedding light on the mechanisms underlying their roles in environmental adaptation and pathogenic processes.

Although significant progress has been made in the study of bacterial MVs, research in this area presents potential challenges due to the complexity of bacterial MVs. Addressing factors such as heterogeneity, contamination, standardization, and functionality is crucial to ensure the reliability and applicability of findings from bacterial MV studies. Firstly, regarding heterogeneity, bacterial membrane vesicles represent a highly heterogeneous population, varying greatly in diameter, content, and membrane composition, requiring complex experimental analyses and theoretical interpretation. Secondly, concerning contamination, bacterial MVs are susceptible to contamination from other extracellular structures such as exosomes, outer membrane vesicles, and protein aggregates, significantly affecting their purity and specificity. Next, concerning standardization, the lack of standardized protocols for the isolation, quantification, and characterization of bacterial MVs may lead to variability and inconsistency in results. Finally, regarding functionality, the functional relevance of bacterial MVs in bacterial physiology and pathogenic mechanisms requires better scientific understanding and explanation, as well as more comprehensive characterization of their effects on host cells and tissues. While it is inevitable to explore the role of MVs in the virulence mechanisms of bacteria, the potential obstacles and limitations of the methods and techniques employed in MV-related functional studies have a significant impact on the interpretation of related results (112).

In current research, most MVs are cultured under standard laboratory conditions, including optimal temperature, air conditions, and abundant nutrient components, and then isolated and purified from large volumes of bacterial culture. Prior to experimental investigations, MVs are typically subjected to thorough concentration and purification validation. However, these methods and steps may overlook the specificity of MV production under microbial stress or stimuli in the natural microbial environment. In the natural environment, bacteria may experience environmental stimuli and stress, differing from bacteria under standard laboratory conditions. Therefore, there may be significant differences in the secretion concentration and composition of MVs produced under laboratory standard culture conditions compared to those produced under microbial stress in the natural environment. Consequently, adopting additional purification methods or appropriate parameter controls to consciously exclude the impact of contaminants other than MVs is crucial for the functional analysis of MVs.

## 5 Summary and prospect

This article provides an overview of the crucial role and current research status of bacterial membrane vesicles (MVs) in environmental adaptation and pathogenic processes (**Table 1**). Recent studies have clearly indicated that many pathogenic microorganisms release significant amounts of virulence factors through MVs, allowing sustained effects without direct contact with host cells (113–116). This characteristic signifies the importance of bacterial MVs in the environmental adaptation and pathogenic processes of pathogenic microorganisms. Bacterial MVs constitute a widely distributed secretion system in Gram-negative bacteria. Research on MVs reveals how bacteria interact with host cells through this secretion pathway, introducing new mechanisms for enhancing bacterial pathogenicity (117). Several studies suggest that the components constituting MVs align with the membrane from which they originate. Mutations in bacterial membrane structure may affect stability and regulate the biological processes of MVs, although mutant strains incapable of producing MVs have not been discovered to date.

**Table 1:** Function of bacteria MVs in environmental adaptation and pathogenic process.

Bacteria	MVs-related Substances	Function of MVs	Refs
*Acinetobacter baumannii*	•TIG-R EV protein	•Tigecycline resistance transfer	(65)
	•Invasive protein antigens:IpaB/IpaC/IpaD	•Invade the host by membrane fusion	
	•Outer membrane proteins: OmpA	•Trigger activation of the host GTPase dynamin-related protein 1 (DRP1)	(81)
*Acinetobacter baylyi*	•Anti-double-stranded DNA (anti-dsDNA)	•Horizontal gene transfer (HGT)	(44)
*Alteromonas macleodii*	•Hydrolytic enzymes	•Facilitate cell wall hydrolysis	(36)
*Bacillus subtilis*	•Virus receptor YueB	•Enhancing the invasion and adsorption of the bacteriophage SPP1	(84)
*Burkholderia cenocepacia*	•Lipopolysaccharides	•Trigger inflammatory response	(106)
*Burkholderia cepacia*	•211 unique proteins •Haemolysin Ahh1 •RtxA toxin •Extracellular lipase •HcpA protein	•Induce the overexpression of proinflammatory cytokines	(98)
*Burkholderia thailandensis*	•Hydroxy-alkyl quinolines (HAQs) •Long-chain rhamnolipids	•Exhibit antimicrobial and anti-biofilm properties •Inhibiting the growth of drug-resistant bacteria and fungi	(67)
*Chromobacterium violaceum*	•Violacein	•Antibiotic action	(66)
*Cupriavidus necator*	•Lipopolysaccharide(LPS)	•Binding effector protein TeoL	(39)
*Dietzia* sp.	•Heme-binding proteins	•Capture and transport heme	(40)
*Edwardsiella piscicida*	•Hemolysins	•Induce caspase-dependent apoptotic-like cell death	(83)
*Escherichia coli*	•β-lactamase (NDM-1)	•Provide protection to nearby bacterial populations	(60)
	•Heat‐labile Enterotoxins	•Mediate entry of active enterotoxin and other bacterial envelope components	(72)
	•Cytotoxic protein cytolysin A (ClyA)	•Secreted toxins	(74, 75)
	•RTX toxin	•Calcium-dependent binding to red blood cells	(76)
	•Anti-tumor cytokines CXCL10 •Interferon-γ	•Antitumor effect	(107)
*Fusobacterium nucleatum*	•Toxic bacterial components	•Polarize macrophages toward an M1-like phenotype	(99)
*Halorubrum lacusprofundi*	•PR1SE encodes proteins	•Infecting plasmid-free strains •Quiring the capacity to produce plasmids	(85)
*Helicobacter pylori*	•Protein Formation	•The difference is significant with the growth stage.	(47)
	•Specific proteins	•Bacterial aggregation and biofilm formation	(90)
	•Cytotoxin-related gene A (CagA) •Vacuolated cytotoxin A (VacA)	•Promote atherosclerosis by inducing pro-inflammatory reaction and plaque formation	(101)
*Klebsiella pneumoniae*	•159 different proteins	•Inducing the expression of pro-inflammatory cytokine genes	(104)
*Lysobacter spp*	•Bacteriolytic enzymes	•Degrade Gram‐positive bacteria	(41)
*Mycobacterium tuberculosis*	•Mycobactin	•Assist in iron intake	(37)
	•FadA •MORN2 •YadA	•Transport virulence factors	(80)
*Myxococcus xanthus*	•Active proteases •Phosphatases •Hydrolases secondary metabolites.	•Kill Escherichia coli cells	(42)
*Neisseria gonorrhoeae*	•Outer membrane porin protein:PorB	•Promoting infection and modulating the cell apoptosis pathway	(102)
*Paracoccus denitrificans*	•N-Hexadecanoyl-L-hoMoserine lactone(C16-HSL)	•Inter-bacterial communication	(51)
*Porphyromonas gingivalis*	•Gingival protease	•Cause damage to the function of host cells	(14)
	•C-terminal domain (CTD) proteins •OmpA peptidoglycan-binding motifs •TonB-dependent receptors	•Transport virulence factors	(71)
	•Proteinfatty acids •Lipopolysaccharide •Peptidoglycan fragments •Nucleic acids	•Induce strong TLR2 and TLR4-specific responses	(105)
*Pseudoalteromonas antarctica*	•Extracellu lar polymeric substance (EPS)	•Deliver proteins to the external media	(58)
*Pseudoalteromonas distincta*	•γ-protein	•Polysaccharide degradation	(33)
*Pseudoalteromonas* sp.	•K-carrageenase	•Degrade the main polysaccharides of red algae cell walls	(35)
*Pseudomonas aeruginosa*	•Pseudomonas quinolone signal (PQS)	•Promote iron bonding	(18, 38)
		•Inhibit biofilm formation of Streptococcus pneumoniae	(95)
		•Inhibit the growth of Staphylococcus aureus	(96)
	•Expression factor:AlgU	•Induce MVs production	(53)
	•Pyocyanin	•Inhibit the growth of other microbial communities	(66)
	•Peptidoglycan hydrolase (autolysin)	•Hydrolyze cell capsule and glycine peptide	(68)
	•β-lactamase •Alkaline phosphatase •Hemolytic phospholipase C •Cif	•Transport virulence factors	(77)
	•EDNAs GlcNAc	•Biofilm formation	(90)
	•Murein hydrolase	•Degrade bacteria cell wall	(49)
	•SRNA52320	•Dampen host immune responses	(97)
*Pseudomonas fluorescens*	•Membrane	•Resist membrane-activeantibiotics	(59)
*Serratia marcescens*	•Anti-malarial protein AmLip	•Targeted killing of plasmodium	(87)
*Staphylococcus aureus*	•α-Toxin	•Red blood cell lysis	(72)
*Streptomyces coelicolor*	•166 kinds of protein •Antibiotics •Vitamins •Amino acids •Carbon metabolism components.	•Participate in cell metabolism/differentiation/molecular processing/transport and stress response.	(32)
*Vibrio cholerae*	•Cholera toxin	•Protect and deliver cholera toxin	(70)
*Vibrio fischeri*	•Outer membrane protein OmpU	•Effective Stimulator for Symbiotic Host Development	(57)
*Vibrio harveyi*	•CAI-1 long chain ketone	•Trigger a QS phenotype	(50)
*Vibrio tasmaniensis*	•Caseinase/gelatinase(VSP)	•Participate in virulence phenotype	(69)
*Yersinia pestis*	•Catalytically active Pla	•Promotes plasminogen activation and α2-antiplasmin degradation	(82)
*Aggregatibacter actinomycetemcomitans/* *Porphyromonas gingivalis/Treponema denticola*	•MsRNAs	•Suppress expression of certain cytokines in Jurkat T cells	(45)
*Haemophilus influenzae/* *Vibrio cholerae*	•Phospholipid transporter protein:VacJ/Yrb	•Deletion leads to the enrichment of phospholipids and fatty acids	(16)

We review various established methods for extracting and analyzing bacterial MVs, emphasizing the indispensability of isolating, quantifying, and characterizing MVs for studying bacterial virulence. Despite significant progress in MV research, challenges persist due to the heterogeneity in size, content, and membrane composition of bacterial MVs, necessitating complex experimental analysis and interpretation. Studies indicate that MVs produced at different growth stages vary in size, protein composition, and immunogenicity, highlighting the importance of considering the bacterial growth stage during MV isolation, as it affects their size, protein composition, and ultimately their biological function. Bacterial MVs can follow different formation mechanisms and cargo sorting mechanisms, resulting in different MV types with distinct biological functions (118). Some published literature may present idealized views, posing significant challenges to the purity and specificity of bacterial MVs due to contamination from extraneous structures such as external bodies and protein aggregates. The lack of standardized protocols for the isolation, quantification, and characterization of bacterial MVs may lead to variability and inconsistency in results. Furthermore, a better scientific understanding of the functional relevance of bacterial MVs in bacterial physiology and pathogenic mechanisms, as well as a thorough characterization of their impact on host cells and tissues, is needed.

Looking ahead, there are several potential applications and future research directions worthy of exploration. Further elucidating the molecular mechanisms of bacterial MVs biogenesis and cargo classification may yield novel targets and strategies for manipulation and regulation. Additionally, understanding the functional relevance and mechanisms of action of bacterial MVs in bacterial physiology and pathogenicity will broaden our understanding of microbial interactions and evolution. Moreover, bacterial membrane vesicles hold broad prospects for biotechnological applications, such as drug delivery, vaccine adjuvants, and diagnostic markers. MVs derived from pathogenic microorganisms can serve as therapeutic targets. Targeting the production or activity of MVs may provide a novel therapeutic strategy for addressing bacterial infections and related diseases (119–121). For instance, inhibiting the biogenesis pathway of MVs has been shown to reduce the transmission of toxic components (122). Based on research into the immunomodulatory effects of MVs, vaccines expressing small non-coding ribonucleic acids could be designed (123), presenting a safe and effective option for cancer treatment with wide-ranging applications. This has significant implications for human health and biotechnology. In conclusion, research on bacterial MVs has expanded our understanding of microbial physiology and pathogenic mechanisms, opening new possibilities for biotechnological and medical applications. Future studies in this field are expected to further unveil the secrets of bacterial MV biogenesis, function, and regulation, with important implications for microbiology, biotechnology, and medicine.

## Author contributions

LX: Software, Visualization, Writing – original draft. YW: Software, Visualization, Writing – original draft. GL: Writing – review & editing. YZ: Writing – review & editing. LH: Funding acquisition, Supervision, Writing – review & editing.
